# Genome-wide mapping of matrix attachment regions in *Drosophila melanogaster*

**DOI:** 10.1186/1471-2164-15-1022

**Published:** 2014-11-25

**Authors:** Rashmi U Pathak, Arumugam Srinivasan, Rakesh K Mishra

**Affiliations:** Centre for Cellular and Molecular Biology, Council of Scientific and Industrial Research, Uppal Road, Hyderabad, 500 007 India

**Keywords:** Nuclear matrix, Matrix attachment regions, Genome packaging

## Abstract

**Background:**

Eukaryotic genome acquires functionality upon proper packaging within the nucleus. This process is facilitated by the structural framework of Nuclear Matrix, a nucleo-proteinaceous meshwork. Matrix Attachment Regions (MARs) in the genome serve as anchoring sites to this framework.

**Results:**

Here we report direct sequencing of the MAR preparation from *Drosophila melanogaster* embryos and identify >7350 MARs. This amounts to ~2.5% of the fly genome and often coincide with AT rich non-coding regions. We find significant association of MARs with the origins of replication, transcription start sites, paused RNA Polymerase II sites and exons, but not introns, of highly expressed genes. We also identified sequence motifs and repeats that constitute MARs.

**Conclusion:**

Our data reveal the contact points of genome to the nuclear architecture and provide a link between nuclear functions and genomic packaging.

**Electronic supplementary material:**

The online version of this article (doi:10.1186/1471-2164-15-1022) contains supplementary material, which is available to authorized users.

## Background

Eukaryotic nucleus is a complex organelle, where DNA is highly compacted but still is accessible for nuclear processes in a precisely controlled manner. The structural basis for such compact but orderly organization is provided by a proteinaceous meshwork known as Nuclear Matrix (NuMat), [1, 2] that has been visualized by electron microscopy [3, 4]. Although existence of NuMat *in vivo* is debated in the context of possible self assembly, this alone is not sufficient to explain all the features of nuclear organization [5]. Non-diffusible fraction of lamin in the interior of the nucleus of living cells indicates the existance of a nucleoskeleton involving such components [6, 7]. The presence of actin, myosin and several cytoskeletal proteins in the nucleus and NuMat further strengthens this view [8, 9]. Whole proteome analysis of NuMat from *Arabidopsis*, *Drosophila* and human have shown that the major categories of proteins these preparations remain the same across species and cytoskeletal proteins are a conserved category [9–11]. NuMat as a framework offers potential basis for compartmentalization of the nucleus and explains why several markers of different nuclear substructures have been found in the NuMat proteome [9]. NuMat is also envisaged as a scaffold on which higher order organization of chromatin loops takes place. Such topologically independent loops define the unit of compartmentalized chromatin that along with differential epigenetic marks bring about proper regulation of gene expression [12, 13].

Biochemically, NuMat consists of DNA, RNA and protein and, while the underlying nuclear structure is not fully characterised, the DNA component belongs to the sequences that help attach the base of chromatin loops to the NuMat [14, 15] and are defined as **M**atrix/**S**caffold **A**ssociated **R**egions, **M/SAR**[16, 17]. Although not much sequence similarity is noticed in different MARs, their biochemical properties have been shown to be conserved [18] presumably due to the secondary structure features and other physical properties of underlying sequences [18–21]. They are nuclease protected regions of chromatin that functionally associate with varitey of *cis*-regulatory elements including origin of replication, chromatin domain boundaries and locus control regions [22–26].

Considering the importance of MARs in genome organization and regulation, a genome–wide map of these sequences is needed to understand the relationship between MAR and known regulatory sequences at genome scale. Efforts have been made in past to experimentally identify number of MARs and to create computational tools to predict them [27, 28]. Here we report, for the first time, mapping and characterization of MARs across the entire 120 Mb of *Drosophila* euchromatic genome. We identify 7353 MARs accounting for ~2.5% of this genome and gives insight into their role in chromatin organization and regulation in the context of genomic packaging.

## Results

### Identification of MAR elements in *Drosophila melanogaster*genome

**Figure 1:**
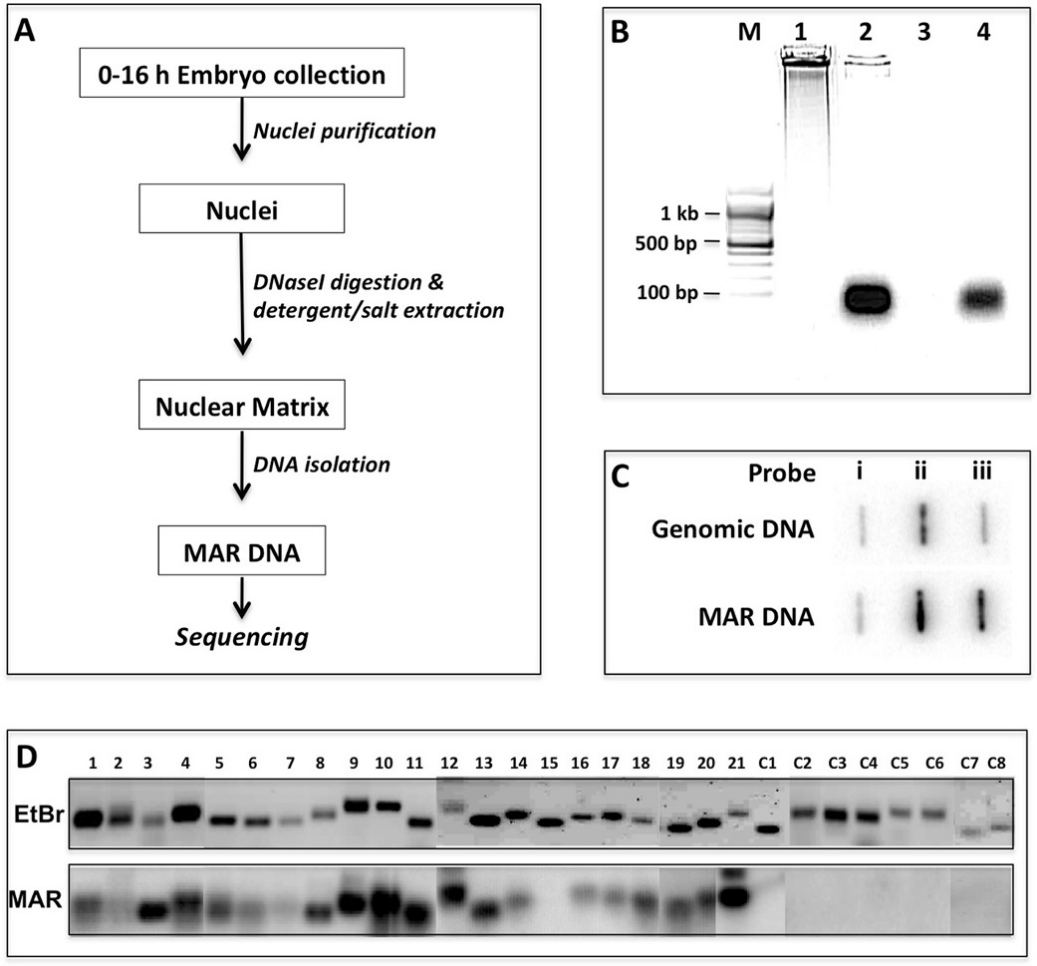
**Isolation and validation of** ***Drosophila*** **embryo MARs. (A)** Overview of MAR DNA isolation procedure. **(B)** Size distribution of MAR DNA after electrophoresis on 1.2% agarose gel. M-Molecular weight marker; Lane 1-Genomic DNA; Lane 2-MAR DNA; Lane 3-MAR DNA + DNaseI; Lane 4-MAR DNA + RNaseA. **(C)** Slot-blot hybridization to show enrichment of known MARs in the MAR DNA preparation. Equal quantity of plasmids (1 μg) carrying sequences of BEAF protein exon as –ve control (slot i), MAR at histone gene locus (slot ii) and scs’ MAR at Hsp70 locus (slot iii) were loaded in each slot. Genomic DNA and MAR DNA were ^32^P labelled by random primer labelling method and used for hybridization of the upper and the lower panel respectively. **(D)** Southern validation for 21 MARs and 8 non-MAR regions chosen on the basis of sequencing data. The upper panel shows the EtBr stained gel profile of the PCR amplified fragments and lower panel shows the blot probed with ^32^P labelled MAR DNA.

**Figure 2:**
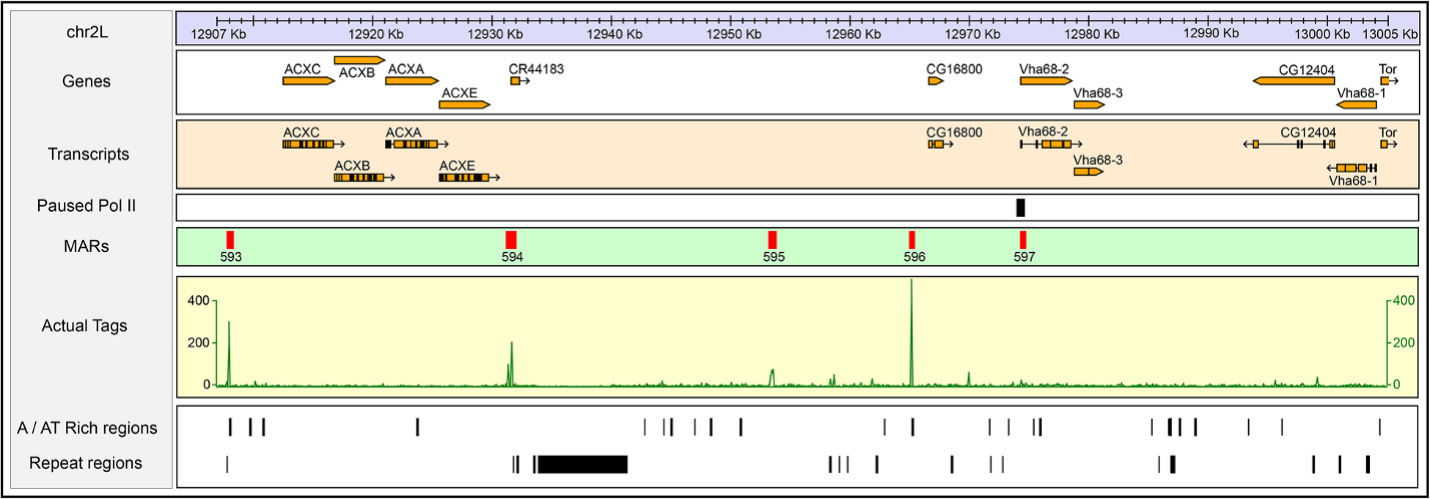
**Distribution of MARs on the ACX and Vha68 gene clusters.** A 98 kb region of chromosome 2 L that encompasses four closely related genes (ACXC, ACXB, ACXA and ACXE) and three genes of Vha68 cluster is drawn to scale. Each boxed sub part shows genes, transcripts, paused pol II, MAR peaks, actual sequence tag counts (xy plot), AT rich regions and repeat regions. The tag counts box shows ‘xy’ plot using the total tag count values of two replicates (100 bp window size). A / AT rich and repeat region tracks are reproduced from the UCSC genome browser.

*D. melanogaster* embryos (0–16 hrs) were collected and used for NuMat preparation using serial extraction steps including DNaseI to digest accessible chromatin DNA (Figure 1A) [29]. Quality of the NuMat preparation was checked using several parameters: retention of 1-2% DNA, ~10% proteins and 30% RNA compared to the nucleus. This preparation was used as source to purify associated genomic fragments as MAR DNA of ~100 bp size that were DNaseI sensitive and RNaseA resistant (Figure 1B). This uniformity in the size of MAR DNA reflects the region that remains in-accessible to the nuclease, while some flanking relevant features may not be retained here. This MAR preparation was further checked for the enrichment of known MARs (Figure 1C) [22, 29]. The MAR DNA preparation was directly sequenced without any further fragmentation. Two biological replicates of MAR preparations were used for direct sequencing that gave a total of 13.8 million mappable reads. The sequenced reads were mapped to the *D. melanogaster* genome (dm3/Release 5) using Bowtie short read mapping algorithm and enriched regions were identified using MACS software (Model based analysis of ChIP seq) [30, 31]. The two replicates were processed independently and gave 13,471 and 13,360 peaks. As shown in Additional file 1: Figure S1, we observed very close similarity in pattern of peaks between the biological replicates. Overall, we found 9215 peaks common between the two replicates that amounts to ~70% of overlap. A windowed (500 bp) comparison of tag counts between the replicates indicates a Pearson’s correlation coefficient of 0.975. This implies that ~97% of the reads from the biological replicates agree with each other. Of the 9215 peaks common to both the biological replicates, we selected 7353 peaks that mapped to euchromatic regions for further analysis, while peaks that mapped to heterochromatic or un-annotated stretches of sequences were left out (Additional file 1: Figure S2)*.* Figure 2 shows a ~100 kb region from chromosome 2 L encompassing ACX and VHA68 gene clusters. Correlation of MARs in the region with AT richness, repeats and paused polymerase II sites is evident.

We randomly picked a few of the identified MARs for validation, before carrying out further analysis. To ascertain that the peaks obtained by sequencing represent true MARs, we performed an *in vivo* MAR-assay with some modification in the original protocol [14]. We used the MAR preparation to make probe and analyzed PCR amplified potential MARs. This modification in the protocol makes it possible to test many candidate sequences on a single Southern blot. We chose 21 MAR and 8 non-MAR sequences with similar AT content for this purpose and, as shown in Figure 1D, almost all of the candidate MARs tested were found to be *in vivo* MARs. Sequences corresponding to negative controls were absent in NuMat preparations.

### MAR prediction tools detect only a fraction of MARs identified in this study

MAR Finder and SMARTest are the two commonly used online tools for prediction of MARs in a given sequence using set of rules related to DNA sequence motifs and conformational features [32, 33]. Large scale, genome or chromosome level, validity of these tools, however, remains to be experimentally established. We find that only 10-15% of the MARs identified in our study are predictable by these *in silico* tools. Vast majority of *in vivo* MARs identified in our study, therefore, are not detectable by existing computational tools. There is also considerable lack of overlap between the output of these two tools. For example, while MAR Finder predicted 1648 MARs on chromosome 3R, SMARTest predicted 3331 MARs, of which only 28.5% were common with the MAR Finder hits. Our analysis gives 1437 MARs in this part of the genome of which only 20.8% were picked by at least one of these tools. Significant improvement is needed to make MAR analysis and prediction tools more effective.

### MAR content and its possible influence on topological feature of genome

**Figure 3:**
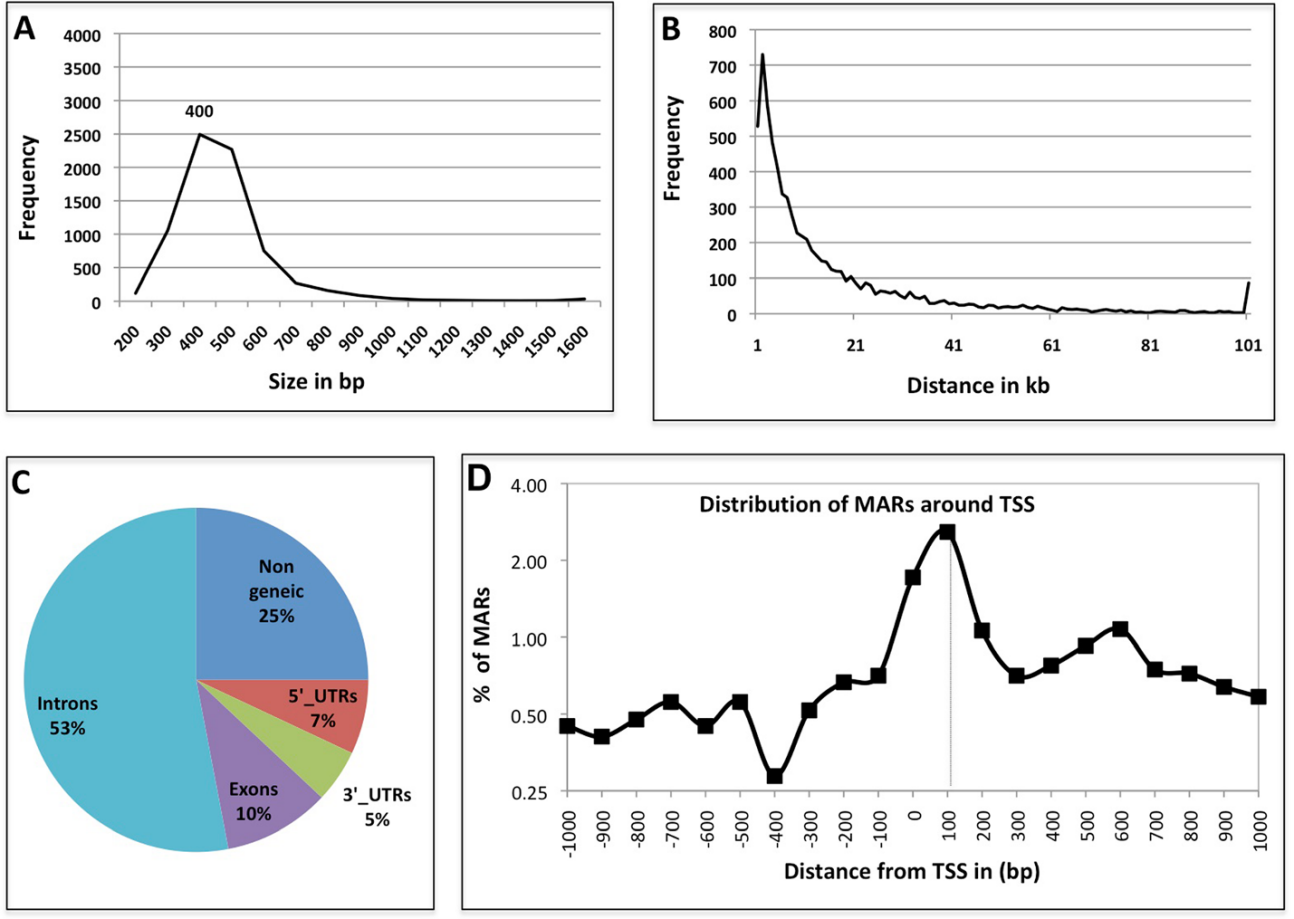
**MAR characteristics. (A)** Size of MARs – in base pairs plotted against their frequency of occurrence in the genome. **(B)** Distribution of inter-MAR distances measured as distance between the midpoints of two contiguous MARs, plotted against their frequency of occurrence in the genome. **(C)** Genomic location of MARs, percentage of MARs localising in various genomic elements. **(D)** MARs occurring from -1 kb to +1 kb of TSS were plotted as a line graph. The graph shows that MARs associate preferentially with paused Pol II site at +100 bp.

MARs identified in this study fall in size range of 0.1-3 kb with a median size of 400 bp. About 90% of the MAR sequences were less than 600 bp long (Figure 3A). Total genomic contribution by the 7353 MARs add up to 3.15 Mb which represents 2.6% of the 120 Mb of euchromatic genome of *Drosophila*. As MARs are associated with higher order organization of chromatin and are expected to hold the base of chromatin loops, we looked into the inter-MAR distance across the genome that may reflect the average chromatin loop-size. We find this distance ranged from <1 kb to 150 kb with an average distance of 16 kb. Overall, 75% of the MARs were less than 20 kb apart (Figure 3B). The data agrees with the reported loop size of 5–200 kb [34]. Our analysis, however, is restricted to the euchromatic region of the genome. The heterochromatic part that contributes up to 30% of the genome, may have different topological features.

### Genomic context of MARs

To find out whether MARs are distributed randomly across the euchromatic regions or have a preferential locations, we looked at the distribution of MARs on different chromosomal arms (Table 1). We observed a distinct increase in MAR density on X chromosome as compared to the autosomes. While, on autosomes we find one MAR for ~20 kb of genomic DNA, the X chromosome has one MAR for ~9 kb DNA. We also noticed gene frequency per MAR on the X chromosome to be ~3 fold less than that on autosome, X chromosome has ~1 gene/MAR while autosomes have ~3 genes/MAR. Since half of the embryo are likely to be male where X chromosome is hyperactive for dosage compensation and active genes associate with MAR [24, 25], this might be a reflection of the higher activity of genes on the X chromosome in male embryos for dosage compensation.

**Table 1:** **Distribution of MARs on chromosomes**

Chromosome arm	Size (Mb)	Number of genes	Gene density (per 100 kb)	Number of MARs	DNA (kb) per MAR	Genes per MAR
2 L	23.0	3048	7.5	1067	21.5	2.9
2R	21.1	3279	6.4	1025	20.6	3.2
3 L	24.5	3001	8.2	1337	18.3	2.2
3R	27.9	3759	7.4	1437	19.4	2.6
4	1.3	100	13.5	34	38.2	2.9
X	22.4	2397	9.4	2453	9.1	1.0
Total	120.4	15584	7.7	7353	16.4	2.1

While 90% of all the MARs are in non-coding regions (Figure 3C) we do find MARs associated with exons, mostly from highly expressed genes like RpLP1, Ef1alpha 48D, Hsc70-3, LamC, Mhc and CG4385 (Additional file 1: Figure S3). 75% of the exonic MARs correlate with moderate to highly expressed genes [35]. This further substantiates the view that MARs have a link to transcriptional activity of the locus associated with them. It remains to be established, however, if structural basis provided by MAR association helps in coupling of transcription and related activity, viz., splicing, or differential epigenetic status and Pol II occupancy of exons/introns [36, 37] results in preferential retention of exons in NuMat. We also noticed that non-genic MARs are smaller compared to exonic ones, average size of 386 bp and 584 bp, respectively, which may reflect different nature or roles of these MARS.

### Analysis of MAR sequences with the known genomic features

#### Association to known MAR features

We looked for the enrichment of DNA sequence features that are reported to be associated with MARs [38]. As shown in Additional file 2: Table S1, ~94% of the MARs follow the ATC rule of one strand having at least a 20 nucleotide stretch of A, T, or C without intervening G, ~95% have Origin of replication (ORI) sequence motifs (ATTA, ATTTA, ATTTTA) and significant proportion of them have features such as curved DNA, AT-rich stretch, etc. Base un-pairing sequences and dTopo II binding sites, however, are missing in most of the MAR sequences identified in this study. The dTopoII binding site used in MAR Finder as well as in our study has been derived from *in vitro* conditions [39]. Later studies show that dTopoII sequence derived *in vitro* was not operative *in vivo*[40] which may explain this apparent inconsistency. However, whether *Drosophila* has different sequence motif for these features and that is why they appear to be missing remains to be ruled out. As SATB1 does not have a homologue in flies, this motif is likely to have got included in MARs later at the time of emergence of vertebrates.

#### Paused polymerase II regions are associated with MARs

We plotted the occurrence of MARs with relation to transcription start sites (TSS). As shown in Figure 3D, most of the MARs around TSS, localize approximately at 100 bp after the TSS. It has been shown that in genes that respond to environmental and developmental cues, Pol II is engaged in early elongation and remains poised at ~50 nucleotides downstream of TSS. Its release into elongation is the rate-limiting step, and stalling Pol II is a way to regulate stimulus response. We find that ~16% of the stalled Pol II regions listed by a study that used 2–4 hrs *Drosophila* embryo, are MARs [41] (Additional file 2: Table S2, Additional file 1: Figure S4). It is probable that stalled Pol II regions are dynamic and many may not remain stalled at later stages of embryonic development and thus do not show up as MARs in our study where 0–16 hrs old embryos were used. This also indicates that the MARs corresponding to paused Pol II regions are dynamic. As this study provides only a snapshot of MARs present in developing embryo, it remains to be seen if these sequences are NuMat associated to cause polymerase stalling or a mere consequence of the process.

#### Enrichment of repeats in MARs

**Figure 4:**
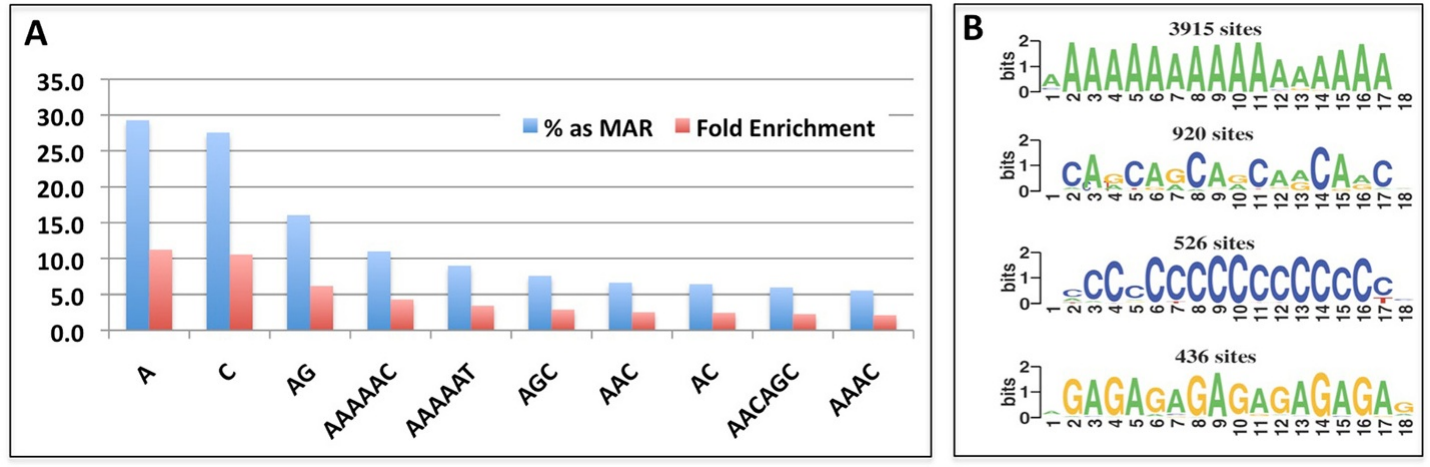
**MAR sequence motifs. (A)** Abundant SSRs present among the MARs are represented as percent of total genomic occurrence and fold enrichment. **(B)** Sequence motifs found to be enriched in MARs based on MuMoD search.

Simple sequence repeats (SSRs) have been proposed to have role in genome packaging and mediation of long-range interactions [42]. We looked for MAR association with SSRs ≥12 bp repeats and present >100 locations in the genome. A subset of SSRs show significant association with MARs (Figure 4A, Additional file 2: Table S3). 29% of A_≥12_ and 27% of C_≥12_ repeats present in euchromatic region are associated with MARs. Among the dimeric repeats, (AG)_6_ and (AC)_6_ were enriched significantly in MARs. About 6% of the trimeric repeats, (AGC)_4_, (ACC)_4_, (AAC)_4_ and (AGG)_4_ are associated with MARs. Among the hexameric repeats, (AAAAAG)_2_, (AAAAAC)_2_ and (AAAAAT)_2_ that closely resemble poly-A stretches with a single mis-match, and (AACAGC)_2_ are enriched in MARs. Interestingly when we looked for novel sequence motifs present in the MARs identified in this study using MuMoD, that uses a Bayesian approach to detect enriched novel DNA motifs without relying on any motifs database, [43] the four most abundant sequence motifs turned out to be SSR related, (A)_12_, (AG)_8_, (AC)_8_ and (AGC)_5_ (Figure 4B). Finally, among transposable elements (TEs) the 5′-untranslated region of the *Drosophila* gypsy retrotransposon has been shown to contain an ‘insulator’ that acts as transcriptional activator of gypsy as well as a MAR/SAR [44]. Thus we looked for enrichment of sequences from TEs in the MARs. We find that sequences from a few retrotransposons are enriched in NuMat (Table 2). From the 49 LTR and 27 LINE-like families of retrotransposons, only 7 show significant enrichment of more than 5 copies being present in the MARs. LINEs show enrichment in the MARs and in most of the cases, the AT-rich 3′-UTR of the TE is a MAR.

**Table 2:** **Transposable elements that associate with NuMat** ^*^

S. No.	TE (size in bp)	TE type	TEs mapping to MARs (%)	MAR size (bp)	Context, location of MAR in TE
1	F-element (4708)	LINE	40	207	3′-UTR, 4483-4689
2	jockey (5154)	LINE	18	105	3′-UTR, 4904-5007
3	Doc (4725)	LINE	67	126	3′-UTR, 4469-4595
4	opus (7521)	LTR	13	291	3′-UTR, 6262-6606
5	gypsy (7469)	LTR	100	127	LTR, 1-126
6	HMS-Beagle (7062)	LTR	45	300	ORF, 831-1130
7	Rt1b (5171)	LINE	36	250	3′-UTR, 4910-5160

*Based on blast 2 sequence comparison of MAR sequence hit region with euchromatic genome sequence (> = 100 bp with > =90% similarity) carried out on Flybase Release 5.48 annotation including partial TEs.

#### MARs as chromatin domain boundaries

As MARs are proposed to be responsible for compartmentalization of genome into functional domains, we looked if these sequences overlap with chromatin domain boundaries. Based on modENCODE ChIP data, two types of boundaries - Class I (occupied by CP190 + CTCF) and Class II [occupied by CP190 + Su(Hw)] were identified earlier [45]. Of the 7353 MARs, only 89 (~1%) overlap with class I and class II boundaries. About 90% of the experimentally determined sites bound with BEAF, CP190, CTCF or Su(HW) do not map to the MARs identified in this study. Along with this, we also looked into a list of predicted boundaries obtained by an *in silico* prediction tool cdBEST, that uses clusters of binding sites of boundary interacting proteins for prediction [46]. Of the cdBEST predicted boundaries, 378 (~5%) overlap with MARs. We, therefore, conclude that only a small subset of MARs function as chromatin domain boundaries. For a closer look, we analysed the *Drosophila* bithorax complex for overlap of boundaries and MARs (Additional file 1: Figure S5). The region has four identified and 11 predicted boundaries. Out of these 15 boundaries only one coincides with one of the thirteen MARs present in this region.

## Discussion

Sequencing of the genome of higher eukaryotes promises insight into the complexity of how genetic information is contained and regulated. To achieve that, however, annotation of various functional elements of the genome needs to be accomplished. Other than protein coding genes, some RNA coding genes and few regulatory elements, much of the genomic content remains to be explored. In the present study, we sequenced MARs from *Drosophila* embryo and mapped to annotate ~2.5% of the euchromatic genome that opens up ways to understand structural basis of genomic packaging and its functional implications.

Sequence analysis of the MARs from our study shows abundance of several known MAR motifs including AT-rich stretches and ORI site. In addition to the known MAR features, our analysis shows enrichment of certain SSRs. Ability of several repeats to influence nucleosome positioning and chromatin level regulatory functions gives structural context of NuMat to these functional repeats [42, 47]. We also found retrotransposon sequences associated with MARs. Transposons are implicated in shaping up of genome architecture, occupy ~5% of euchromatic genome and many occurrences are restraint by transcriptional inactivation [48]. While functional link between MAR and retrotransposons remains be investigated, our findings and earlier report that gypsy retrotransposon, which functions as chromatin insulator element, is also associated with nuclear matrix [44], provide possible structural basis to it.

MARs link the genomic loci to the nuclear architecture and by that are proposed to define topologically independent domains. NuMat proteins that bind to DNA may have a role in the process. For example, Lamin B, an abundant structural protein in the NuMat has been shown to bind to S/MARs [49]. We earlier identified 354 NuMat proteins from *Drosophila* embryo, where ~13% were related to DNA binding or chromatin remodelling and, possibly, bridge NuMat to chromatin *in vivo*[9]. We find an average inter-MAR distance and, thereby, chromatin loop size to be 16 kb, which supports the earlier reports showing the loops in active chromatin regions to range from 5 to 13 kb [14, 34]. This also raises the possibility of a cohabitation of chromatin domain boundary and MAR in agreement with the mechanism where boundaries interact with nuclear lamina, nucleolus, and NuMat as well [50]. However, we found only ~5% overlap between boundaries and MARs. This shows that most of the boundaries do not associate with NuMat and insulator bodies may be a NuMat independent feature. It remains, however, to be formally ruled out if only a subset of boundaries are functional in a given cell type and partial overlap with MAR reflects that.

We find several MARs in coding regions that are actively transcribed or are poised for transcription. It has been shown that association of chromosomes with NuMat determines its transcriptional activity [51, 52]. We expect transcription related MARs to be dynamic and dependent on the transcription profile of the cell in question, although it remains to be firmly established. Interestingly, we find that X chromosome, that is known to be hyper-activated in *Drosophila* males for dosage compensation, has double the MAR density compared to the rest of the genome. A direct link between MAR and dosage compensation remains to be explored. Another striking feature, in the context of transcription and MAR emerged as the association of stalled Pol II promoters with MAR [41, 53, 54]. This indicates that transcriptionally engaged Pol II accumulates just downstream of the promoters and structural basis for this state is provided by the association with NuMat. In the actively transcribed genes, we also noticed that only exons were associated with NuMat. It has become increasingly clear that transcription and splicing are coupled events as both the processes are executed in NuMat in a co-ordinated manner. It has also been shown that Pol II accumulates mostly at exons during transcription and only exons are tethered to Pol II transcription machinery excluding introns by looping out [55]. Such an arrangement helps in accurate splice site selection especially across large introns that may have many alternative splice sites [36]. As Pol II transcription machinery is closely associated with NuMat, enrichment of exonic sequences in MARs provides the structural link between the two.

We also noticed that the average MAR size measures to 2–3 nucleosomal space. This incidentally is the number of nucleosomes that get displaced during many nuclear processes like transcription, and double stranded break repair [56, 57]. As these processes are executed in close proximity of NuMat, MARs of this size may be the reflection of functional DNA in action at a given instance. MARs in coding regions were, however, relatively longer than those in non-coding regions. While the relevance of this remains to be investigated, this observation is in agreement with earlier report of a similar difference in size of MARs from coding and non-coding regions that was observed in HeLa cell [28].

We observed very little overlap between the output of MAR prediction tools as well as the MARs identified in this study. One possibility is that various ‘rules’ may not have been given appropriate weightage or these rules apply less to *Drosophila* genome. While further studies will be required to settle this issue, MAR prediction tools will need to incorporate functional/epigenetic state of the region and its dynamic nature among the parameters. Several observations in our study indicate functional links of MAR, viz., association with exons of highly expressed genes, longer MARs from exons and association with sites of paused RNA Pol II which adds conditional MAR situation in addition to generally considered facultative MARs.

## Conclusions

In conclusion, mapping of nuclear matrix attachment regions across 120 Mb of euchromatic *Drosophila* genome reveals strong link between MAR and transcriptional status of the locus. We also find MARs cohabiting with regulatory functions, viz., ORI sequences, paused Pol II sites, domain boundaries, etc. These observations reflect the structural perspective of nuclear architecture in context of functioning of these elements. It is also possible that there are two kinds of MARs, one that function as structural elements – attach genome to the nuclear matrix, for example, to define chromatin loops and the other that associate with components of nuclear architecture as a consequence being part of a regulatory mechanism, for example, pausing of transcription. While the first kind is expected to be constant or static MAR, the second one is likely to be transient or dynamic which will depend on cell type or functional state of the associated loci. Our study leads to a better understanding of the elements that define the chromatin landscape and co-ordinate packaging and regulation of genome in nuclear space. Mapping of MARs at genome scale offers a structural look at the high throughput epigenetic modifications now available in public domain and an understanding of the structural basis of epigenetic regulation of gene expression. Further studies will be needed to examine such regulatory elements in the context of specific cell types.

## Methods

### MAR DNA preparation from *Drosophila*embryos

Embryos (0–16 hr) were collected from a laboratory strain of *Drosophila melanogaster* (Canton-S) maintained at 25°C. All the chemicals used for MAR DNA isolation were obtained from Sigma-Aldrich (St Louis, MO) unless mentioned otherwise. NuMat was prepared according to published protocol [14] with modifications as specified below. Figure 1A gives the overview of the MAR DNA isolation procedure. Briefly, nuclei were isolated from 1 gm of 0–16 hrs *Drosophila* embryos. An aliquot of nuclei was stored for isolation and estimation of total genomic DNA for quality control checks. To prepare NuMat, chromatin was removed by DNaseI digestion in a buffer containing 20 mM Tris pH 7.4, 20 mM KCl, 70 mM NaCl, 10 mM MgCl_2_, 0.125 mM spermidine 1 mM PMSF, 0.5% Triton-X 100, and 200 μg/ml DNaseI (Sigma) at 4°C for 1 hr. Chromatin depleted nuclei were collected by centrifugation at 3000xg for 10 min. Digestion was followed by extraction with 0.4 M NaCl for 5 min in extraction buffer containing 10 mM Hepes pH 7.5, 4 mM EDTA, 0.25 mM spermidine, 0.1 mM PMSF, 0.5% (v/v) Triton X-100 and another 5 min with 2 M NaCl in the extraction buffer. The final pellet after extraction was washed twice with wash buffer (5 mM Tris pH 7.4, 20 mM KCl, 1 mM EDTA, 0.25 mM spermidine, 0.1 mm PMSF). To remove RNA, RNaseA was added to a final concentration of 20 μg/ml. Incubation was carried out at 37°C for 30 min to remove all associated RNA. This was followed by digestion with 100 μg/ml Proteinase K at 55°C for 1 hr. DNA was recovered by extraction with phenol:chloroform:isoamyl alcohol (25:24:1) and ethanol precipitation. Precipitated DNA was dissolved in water and quantitated by measuring the absorbance at 260 nm. We checked the quality of DNA obtained from respective fractions on an agarose gel (Figure 1B). We always found the size of the MAR DNA obtained to be less than 100 bp (Figure 1B, Lane 2). The MAR DNA was susceptible to DNaseI and resistant to RNaseA, confirming its identity as DNA (Figure 1B, Lane 3, 4). On estimation we found that ~1% of the total nuclear DNA was retained in NuMat preparation. These figures were fairly constant and we used it as a quality check for assessing our MAR DNA preparations.

### Sequencing of MAR DNA

All the kits and reagents used were obtained from Applied Biosystems (Life Technologies). The MAR DNA sequences were already in the range of 50–100 bp and so were used straight away to prepare fragment library according to manufacturer’s instruction using Fragment Library Construction Kit. Libraries were prepared from two independent biological replicates of 0–16 hrs *Drosophila* embryos. Briefly, 5 μg of DNA was end-repaired, ligated to P1 and P2 adaptors, and size selected on a gel where 50–100 bp fragments were selected. Using the adaptors, the size-selected DNA was amplified for 3 cycles to generate the fragment library. Library was quantified using the Agilent 2100 Bioanalyser (Agilent Technologies Inc.). Templated beads were prepared according to manufacturer’s instructions using ePCR kit and the Bead Enrichment Kit. A Workflow Analysis was done using the Workflow Analysis Kit, to estimate that a sufficient number of good quality templated beads were produced. Templated beads were deposited on a slide using Bead Deposition Kit. Beads from one library were deposited on a quad of a slide, hence one slide carried libraries of the two replicates. The slide was run on an ABI SOLiD v2 Sequencer according to the manufacturer’s instruction.

### Validation of identified MARs by *in vivo*MAR assay

To acertain that the sequences identified as MARs by SOLiD sequencing were true representation of MARs present in *Drosophila* nuclei, we performed an *in vivo* MAR assay that was modified from the original protocol as described in Mirkovitch et al. 1984. [14] Twenty nine sequences chosen from all arms of the chromosomes were PCR amplified from *Drosophila* genomic DNA using primers listed in Additional file 2: Table S4. Of the 29 candidate sequences tested, 21 were identified as MARs by SOLiD sequencing. Eight sequences that were not MARs, were chosen as negative controls. The amplified fragments were resolved on an 1.2% TAE agarose gel and transferred to charged Nylon membrane using capillary transfer method. Total MAR DNA isolated in exactly the same way as detailed above and used for SOLiD sequencing, was labelled with ^32^P-dATP by Random Primer Labelling method. Southern hybridization was carried out at 55°C for 16 hours after which the blots were washed stringently and imaged. The PCR amplified fragments that were MARs *in vivo*, hybridised to corresponding ^32^P – labeled fragments from the total MAR DNA preparation and showed signal. The negative controls, as expected, did not show any ^32^P signal. The present protocol is a reversed version of the original one, where the total MAR DNA is Southern blotted and probed with candidate ^32^P-labeled probes individually. The modification helped us in interrogating 29 candidate sequences with the same ^32^P – labeled total MAR DNA probe on a single blot.

### Data processing and analysis

We trimmed all sequenced tags to 24 bp and mapped the tags to the *D. melanogaster* genome (dm3, BDGP Release 5) using Bowtie v0.12.7 algorithm with command-line option ‘-v 3’. Each experimental replicate was handled separately. To calculate the Pearson-correlation between the replicates, we counted the number of tags for each available 500 bp window across the genome and compared them between the replicates using a Perl script. Peaks were called for each experimental replicate separately using MACS (v1.4.0rc2) tool with the options ‘--pvalue 0.001, --llocal =50000’. The common-peak regions that have at least 50 bp overlap between replicates were extracted. These common peak regions and their sequences alone were used for the subsequent data analysis. 7353 MAR peaks mapped to euchromatic region are listed in a ‘BED file’ are shown in Additional file 3. To categorize the MARs as genic and non-genic, the midpoints of MAR regions were compared with gene annotation data (FlyBase r5.48). The MARs that fell on genic regions were further sub-categorized as 5′_UTR, 3′_UTR, exonic and intronic MARs. The stalled and active Pol II gene promoter coordinates (±300 bp of TSS) were obtained using Zeitlinger J et al. [41] and directly compared them with MAR regions to find an association. Boundary element sequence coordinates were retrieved from available literature [46, 58]. A set of known MAR signal/motifs [32] were used for querying our MAR data set to map their occurrences. SMARTest (euchromatic portion) and MAR-WIZ (chr3R) tools were used for *in silico* MAR predictions [32, 33]. To find the SSRs that are enriched in MARs, we used the 501 non-redundant SSRs combinations [59] and a custom written Perl script. Minimum of 12 bases was considered to pick as SSR occurrence. MARs sequences were aligned with Transposable Elements (Flybase) using the locally installed NCBI’s “bl2seq” tool (release 2.2.22). Sequence similarity of ninety percent over a 100 bp sequence segment was used as criterion to call aligned hits.

### Data access

The sequence data from this study has been submitted to the NCBI Sequence Read Archive under accession number SRX443533.

## Electronic supplementary material

Additional file 1: Figure S1: Replicates of MAR samples highly agree with each other. **Figure S2** – Distribution of MARs across chromosomal arms. **Figure S3 –** Exonic MARs strongly correlate with high level of expression. **Figure S4 –** MARs overlap with paused PolII promoter regions. **Figure S5 –** MAR and chromatin domain boundary don’t always coincide. (PDF 11 MB)

Additional file 2: Table S1: Enrichment of known MAR_DNA features. **Table S2 –** MAR sequences overlapping with stalled PolII regions. **Table S3 –** SSRs in MAR sequences. **Table S4 –** Primers used for amplification of MARs. (PDF 219 KB)

Additional file 3: **BED file –** **List of all MARs mapped to the euchromatic region.** (ZIP 82 KB)

## Abbreviations

NuMat: Nuclear Matrix
M/SARs: Matrix/Scaffold attachment regions
TSS: Transcription start site
ORI: Origin of replication
SSRs: Simple sequence repeats
TE: Transposable elements.
