# Supplementary material for: Genome-wide mapping of matrix attachment regions in Drosophila melanogaster
Source: BMC Genomics. 2014 Nov 25;15(1):1022. doi: 10.1186/1471-2164-15-1022 (PMC4301625; doi:10.1186/1471-2164-15-1022)
Supplement: Supplementary file 1 — Additional file 1: Figure S1: Replicates of MAR samples highly agree with each other. Figure S2 – Distribution of MARs across chromosomal arms. Figure S3 – Exonic MARs strongly correlate with high level of expression. Figure S4 – MARs overlap with paused PolII promoter regions. Figure S5 – MAR and chromatin domain boundary don’t always coincide. (PDF 11 MB) [file 12864_2014_6839_MOESM1_ESM.pdf]

Supplementary Figure 1

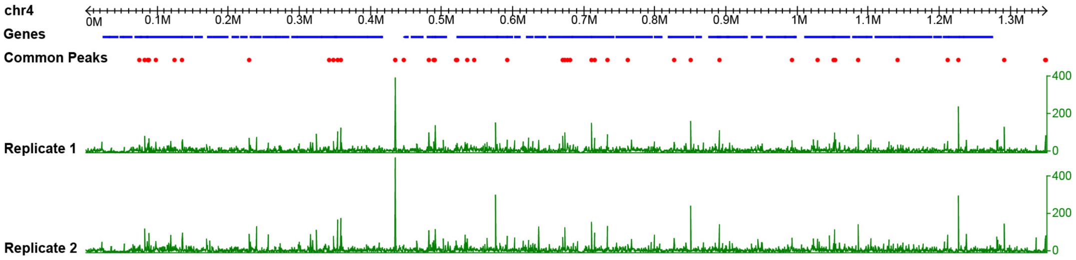

Supplementary Figure 1. Replicates of MAR samples highly agree with each other.
Actual tag counts (500bp window) of biological replicates are shown as green xy plot for the entire length of chromosome 4. In the y axis the scale is shown upto 400 tags. The MAR peaks that are common to both replicates are shown as red dots and the genes are shown as blue bars according scale (Mega bases).

Supplementary Figure 2.

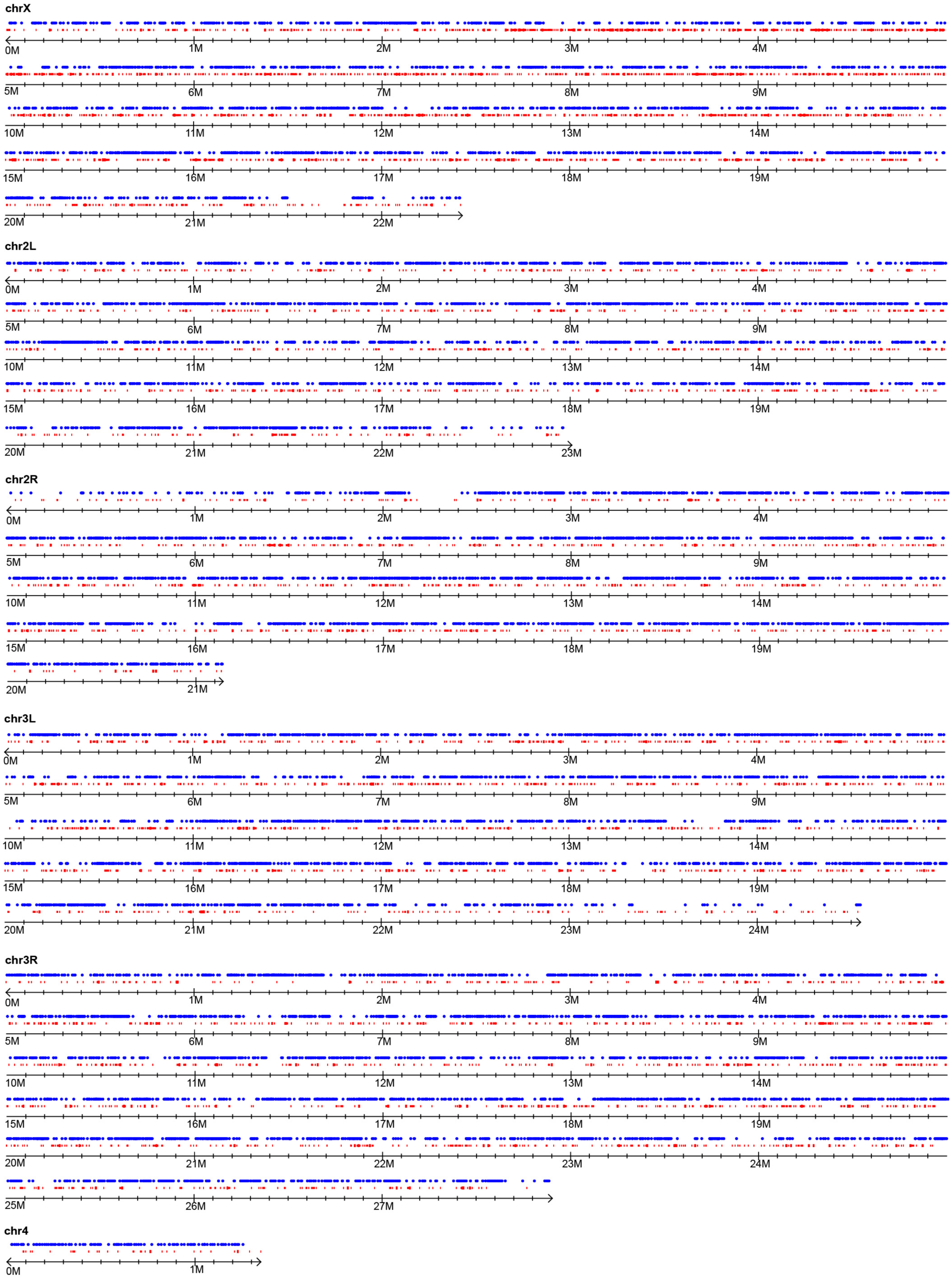

Supplementary Figure 2. Distribution of MARs across chromosomal arms.
MAR peaks are shown as red boxes and the genes are shown as blue dots according scale (Mega bases).

Supplementary Figure 3

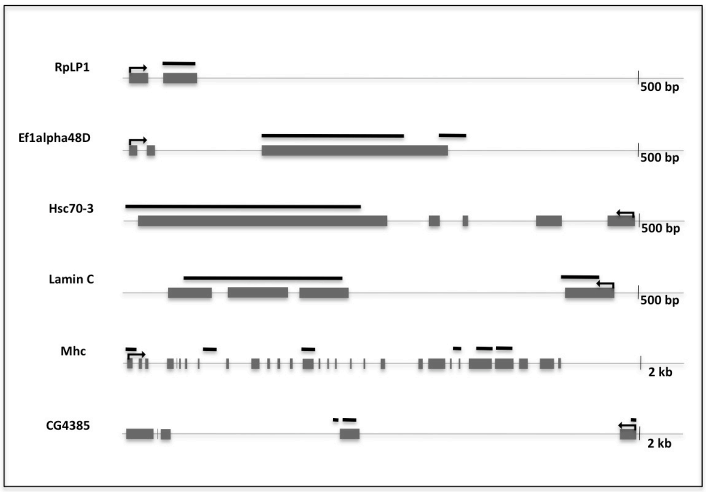

Supplementary Figure 3. Exonic MARs strongly correlate with high level of expression.
UCSC Genome Browser images of a few highly expressed genes with introns (grey lines) and exons (grey bars) are shown. The black bars indicate the MARs.

Supplementary Figure 4

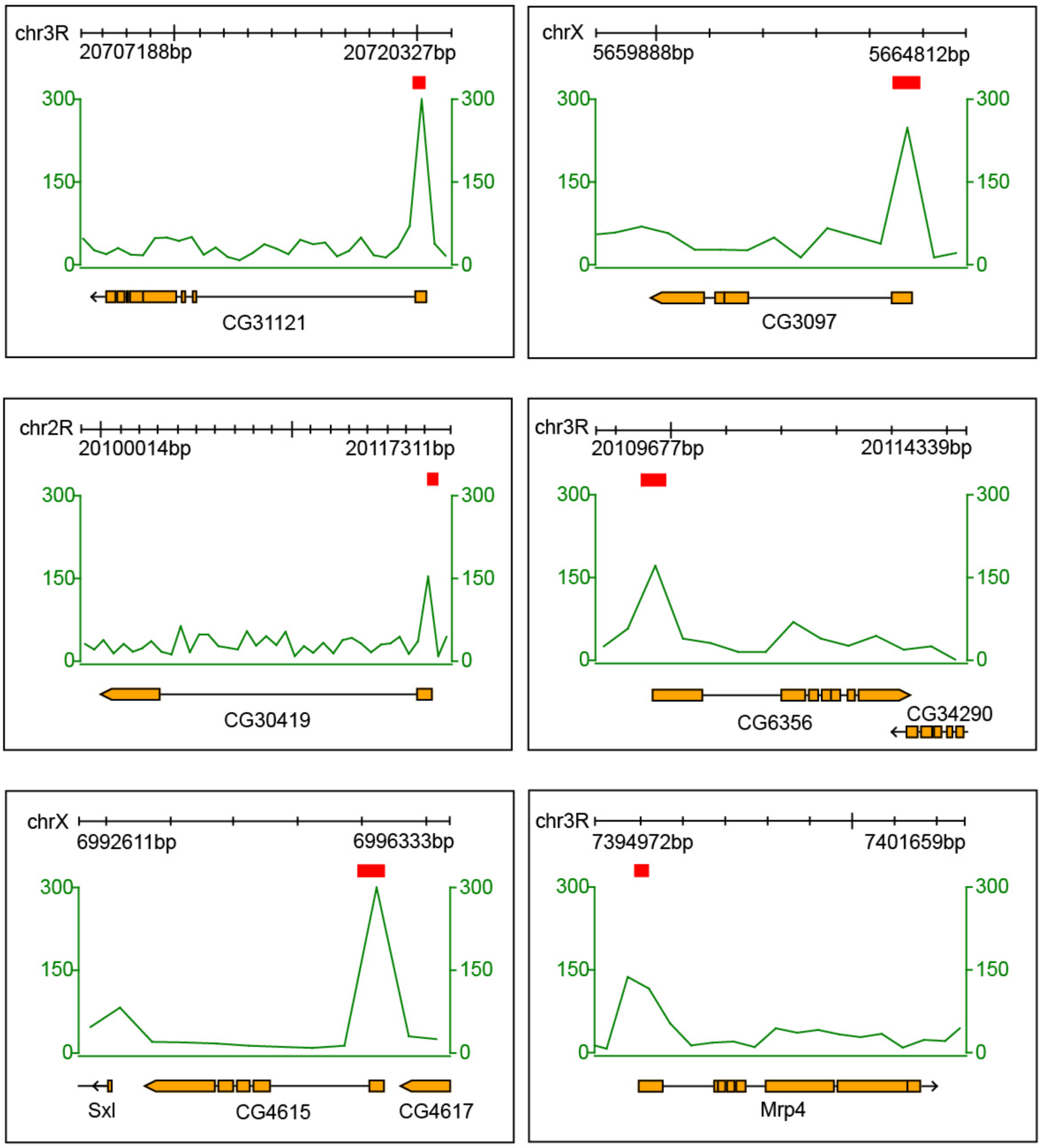

Supplementary Figure 4. MARs that overlap with paused Pol II promoter regions.
Few genes with stalled Pol II promoters are drawn to scale. The MARs are shown as red bars. Actual tag counts (500bp window) are shown as green xy plot for the entire length of gene.

Supplementary Figure 5

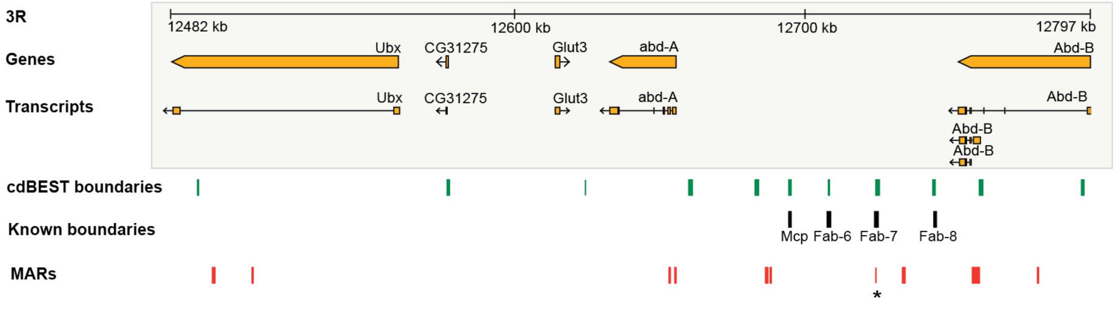

Supplementary Figure 5. MAR and chromatin domain boundary don't always coincide. The grey upper panel shows 315kb BX-Complex region of 3R chomosome with genes and transcripts. The known and cdBEST boundaries are shown as black and green vertical bars. MARs are shown as red bars. The asterisk indicates the MAR that coincides with Fab-7 boundary.
