# Supplementary material for: Genome-wide mapping of matrix attachment regions in Drosophila melanogaster
Source: BMC Genomics. 2014 Nov 25;15(1):1022. doi: 10.1186/1471-2164-15-1022 (PMC4301625; doi:10.1186/1471-2164-15-1022)
Supplement: Supplementary file 2 — Additional file 2: Table S1: Enrichment of known MAR_DNA features. Table S2 – MAR sequences overlapping with stalled PolII regions. Table S3 – SSRs in MAR sequences. Table S4 – Primers used for amplification of MARs. (PDF 219 KB) [file 12864_2014_6839_MOESM2_ESM.pdf]

Supplementary Table 1: Enrichment of known MAR_DNA features

S. No.	Feature	Associated Motif*	In % of MARs†
1	Origin of replication	ATTA, ATTTA, ATTTTA	94.7
2	H-rule/ATC rule	H_{20}	93.5
3	AT-Rich Signal	$W_6N_{8-12}W_6$	74.3
4	TG-rich signal	TGTTTTG, TGTTTTTTG, TTTTGGGG	29.2
5	Curved DNA	$A_4N_7A_3N_7A_4$, $T_4N_7T_3N_7T_4$, TTTAAA	51.0
6	Kinked DNA	TAN_3TGN_3CA, TAN_3CAN_3TG, TGN_3TAN_3CA, TGN_3CAN_3TA, CAN_3TAN_3TG, CAN_3TGN_3TA	30.3
7	Base un-pairing sequence	AATATATTT	1.7
8	dTopo binding site	GTNWAYATTNATNNR	0.5
9	MRS Signature	AATAAYAA, AWWRTAANNWWGNNNC	15.0

* Motif sequences are defined as IUPAC code for DNA. The subscript numbers indicate stretch of a nucleotide that is required.

† The percentage of MARs that are having one or more motifs in the subset.

Supplementary Table 2. MAR sequences overlaping with stalled Pol II regions

S. No.	Gene	MAR_ID	Chromosome	Start	Stop
1	Gs1	em_common_peak-2	chr2L	131966	132350
2	cbt	em_common_peak-15	chr2L	476775	479793
3	drongo	em_common_peak-31	chr2L	850422	850804
4	kraken	em_common_peak-33	chr2L	852790	853154
5	S	em_common_peak-46	chr2L	1077536	1077887
6	ast	em_common_peak-46	chr2L	1077536	1077887
7	CG4710	em_common_peak-48	chr2L	1118740	1119237
8	CG4726	em_common_peak-49	chr2L	1142970	1143465
9	CG31666	em_common_peak-70	chr2L	1651139	1651496
10	CG18317	em_common_peak-81	chr2L	1723812	1724320
11	CG7337	em_common_peak-86	chr2L	1885009	1885305
12	GlyP	em_common_peak-92	chr2L	2130756	2131521
13	aop	em_common_peak-93	chr2L	2161365	2161900
14	Slh	em_common_peak-99	chr2L	2492896	2493181
15	oaf	em_common_peak-99	chr2L	2492896	2493181
16	CG2991	em_common_peak-119	chr2L	2862478	2862830
17	NTPase	em_common_peak-120	chr2L	2884972	2885281
18	CG3523	em_common_peak-130	chr2L	3056401	3056884
19	Thor	em_common_peak-151	chr2L	3478252	3479586
20	CG3714	em_common_peak-190	chr2L	4196840	4197226
21	tutl	em_common_peak-193	chr2L	4282966	4283393
22	Atet	em_common_peak-199	chr2L	4333779	4334214
23	CG15628	em_common_peak-221	chr2L	4821348	4821558
24	pgant5	em_common_peak-235	chr2L	5093355	5093601
25	Gpdh	em_common_peak-262	chr2L	5943750	5944166
26	Pez	em_common_peak-283	chr2L	6338777	6339149
27	Cpr	em_common_peak-283	chr2L	6338777	6339149
28	nrv1	em_common_peak-294	chr2L	6786554	6787039
29	Wnt4	em_common_peak-319	chr2L	7276797	7277380
30	CG7134	em_common_peak-340	chr2L	7810104	7810476
31	SoxN	em_common_peak-381	chr2L	8825559	8825961
32	Trx-2	em_common_peak-412	chr2L	9613339	9613660
33	Pka-C1	em_common_peak-416	chr2L	9698983	9699364
34	Myo31DF	em_common_peak-464	chr2L	10506512	10506828
35	CG17124	em_common_peak-483	chr2L	10755995	10756294
36	Jhl-21	em_common_peak-539	chr2L	12055669	12056054
37	Vha68-2	em_common_peak-597	chr2L	12974297	12974669
38	B4	em_common_peak-617	chr2L	13548818	13549442
39	CG9008	em_common_peak-636	chr2L	13779882	13780262
40	smi35A	em_common_peak-663	chr2L	14233914	14234239

41	elB	em_common_peak-672	chr2L	14409307	14409594
42	Gli	em_common_peak-737	chr2L	15762491	15762888
43	Cyt-c-d	em_common_peak-792	chr2L	16715857	16716252
44	beat-IIIc	em_common_peak-818	chr2L	17260484	17260802
45	CG6860	em_common_peak-822	chr2L	17383873	17384166
46	Socs36E	em_common_peak-853	chr2L	18152151	18152470
47	Fas3	em_common_peak-855	chr2L	18319921	18320380
48	CG17321	em_common_peak-882	chr2L	18844970	18845493
49	CG10641	em_common_peak-893	chr2L	18986962	18987347
50	drl	em_common_peak-912	chr2L	19190260	19190646
51	TepIV	em_common_peak-929	chr2L	19549489	19549944
52	Hr39	em_common_peak-995	chr2L	21237090	21237429
53	Mio	em_common_peak-997	chr2L	21309233	21309641
54	CG9397	em_common_peak-1130	chr2R	2389436	2389653
55	Rab2	em_common_peak-1137	chr2R	2584415	2585156
56	Tsp42Ef	em_common_peak-1145	chr2R	2912341	2912796
57	Dscam	em_common_peak-1158	chr2R	3269236	3269600
58	CG1600	em_common_peak-1161	chr2R	3416382	3416869
59	CG8713	em_common_peak-1188	chr2R	3979128	3979507
60	Rab-RP1	em_common_peak-1227	chr2R	5073707	5074016
61	ced-6	em_common_peak-1239	chr2R	5328772	5329161
62	CG1623	em_common_peak-1255	chr2R	5720635	5721079
63	CG12214	em_common_peak-1269	chr2R	6045808	6046294
64	CPTI	em_common_peak-1282	chr2R	6362749	6363144
65	CG30015	em_common_peak-1317	chr2R	6761985	6762384
66	CG8964	em_common_peak-1381	chr2R	7912574	7912960
67	jeb	em_common_peak-1386	chr2R	8006523	8006970
68	Oda	em_common_peak-1388	chr2R	8056133	8056857
69	Cam	em_common_peak-1396	chr2R	8146835	8147193
70	chn	em_common_peak-1566	chr2R	11016225	11016529
71	Fs	em_common_peak-1578	chr2R	11128900	11129231
72	Arf51F	em_common_peak-1580	chr2R	11209653	11209949
73	CG8180	em_common_peak-1590	chr2R	11370102	11370393
74	CG8253	em_common_peak-1609	chr2R	11736054	11736652
75	Dg	em_common_peak-1621	chr2R	11985922	11986129
76	spin	em_common_peak-1623	chr2R	12012434	12012617
77	CG30463	em_common_peak-1668	chr2R	12618707	12619099
78	GstS1	em_common_peak-1685	chr2R	12984690	12985191
79	grh	em_common_peak-1729	chr2R	13711021	13711445
80	edl	em_common_peak-1775	chr2R	14560704	14561422
81	Glycogenin	em_common_peak-1914	chr2R	17087890	17088298
82	CG18375	em_common_peak-1918	chr2R	17138228	17138599
83	CG6437	em_common_peak-1971	chr2R	17963386	17964021
84	dve	em_common_peak-1976	chr2R	18158241	18158728
85	wdp	em_common_peak-1979	chr2R	18199192	18199504

86	CG13506	em_common_peak-1983	chr2R	18270433	18270929
87	CG33143	em_common_peak-2001	chr2R	18501789	18502127
88	jbug	em_common_peak-2012	chr2R	18616525	18616882
89	nahoda	em_common_peak-2017	chr2R	18783580	18783962
90	CG9896	em_common_peak-2019	chr2R	18886156	18886512
91	CG11299	em_common_peak-2049	chr2R	19601240	19601761
92	CG30419	em_common_peak-2067	chr2R	20117176	20117661
93	zip	em_common_peak-2087	chr2R	20898674	20899030
94	CG13893	em_common_peak-2131	chr3L	603821	604161
95	CG13894	em_common_peak-2140	chr3L	698842	699242
96	CG32333	em_common_peak-2170	chr3L	1275862	1276215
97	rho	em_common_peak-2182	chr3L	1463400	1464055
98	cue	em_common_peak-2190	chr3L	1517032	1517360
99	Psa	em_common_peak-2190	chr3L	1517032	1517360
100	CG13921	em_common_peak-2195	chr3L	1746753	1747231
101	CG15822	em_common_peak-2214	chr3L	2163675	2163904
102	Shab	em_common_peak-2258	chr3L	2894848	2895552
103	scrt	em_common_peak-2326	chr3L	3983987	3984284
104	CG14998	em_common_peak-2333	chr3L	4134034	4134499
105	nab	em_common_peak-2335	chr3L	4160824	4161309
106	Ero1L	em_common_peak-2337	chr3L	4170248	4170362
107	ImpL2	em_common_peak-2340	chr3L	4226884	4227425
108	CG18314	em_common_peak-2346	chr3L	4380113	4380627
109	lin-28	em_common_peak-2432	chr3L	5647661	5648019
110	vvl	em_common_peak-2489	chr3L	6783202	6783536
111	sgl	em_common_peak-2508	chr3L	6957480	6958027
112	CG33556	em_common_peak-2516	chr3L	7087741	7088223
113	CdsA	em_common_peak-2601	chr3L	8121151	8121753
114	ImpE1	em_common_peak-2611	chr3L	8365133	8365483
115	h	em_common_peak-2634	chr3L	8668700	8669143
116	CG32041	em_common_peak-2674	chr3L	9367137	9368094
117	Hsp26	em_common_peak-2675	chr3L	9369289	9370771
118	Hsp23	em_common_peak-2676	chr3L	9375000	9375854
119	LanB2	em_common_peak-2694	chr3L	9607946	9608276
120	CG32062	em_common_peak-2747	chr3L	10509024	10509360
121	CG32066	em_common_peak-2766	chr3L	10653852	10654165
122	simj	em_common_peak-2767	chr3L	10657788	10658101
123	tna	em_common_peak-2789	chr3L	10850823	10851677
124	klu	em_common_peak-2806	chr3L	11001990	11002371
125	CG32082	em_common_peak-2810	chr3L	11129034	11129359
126	CG18490	em_common_peak-2815	chr3L	11208664	11208987
127	chrb	em_common_peak-2841	chr3L	11480495	11481420
128	CG6083	em_common_peak-2853	chr3L	11619229	11619534
129	CG11658	em_common_peak-2856	chr3L	11701386	11701765
130	CG32105	em_common_peak-2885	chr3L	12321646	12322049

131	CG32111	em_common_peak-2905	chr3L	12620787	12621271
132	CG32130	em_common_peak-2948	chr3L	13470454	13471907
133	bru-3	em_common_peak-2965	chr3L	13659438	13659974
134	btl	em_common_peak-2985	chr3L	14070140	14070403
135	fz	em_common_peak-2990	chr3L	14267377	14267705
136	CG9238	em_common_peak-2998	chr3L	14543937	14544224
137	ome	em_common_peak-3004	chr3L	14699658	14700032
138	Toll-6	em_common_peak-3033	chr3L	15329899	15330269
139	CG6498	em_common_peak-3044	chr3L	15558468	15558881
140	comm	em_common_peak-3054	chr3L	15721247	15721337
141	Baldspot	em_common_peak-3110	chr3L	16654379	16654762
142	Galpha73B	em_common_peak-3111	chr3L	16660876	16661248
143	Eip75B	em_common_peak-3178	chr3L	17992339	17992773
144	CG6896	em_common_peak-3196	chr3L	18618975	18619259
145	CG32227	em_common_peak-3289	chr3L	20288045	20288453
146	CG4786	em_common_peak-3296	chr3L	20533275	20533689
147	fng	em_common_peak-3316	chr3L	20942523	20942812
148	Eip78C	em_common_peak-3337	chr3L	21233404	21233764
149	CG33291	em_common_peak-3349	chr3L	21637934	21638299
150	mub	em_common_peak-3353	chr3L	21872598	21872897
151	olf413	em_common_peak-3361	chr3L	22129104	22129439
152	CG11739	em_common_peak-3436	chr3R	204442	204939
153	CG31531	em_common_peak-3442	chr3R	485193	485605
154	Cdep	em_common_peak-3460	chr3R	736093	736606
155	corto	em_common_peak-3471	chr3R	912092	912847
156	lab	em_common_peak-3512	chr3R	2503632	2504057
157	CG8036	em_common_peak-3595	chr3R	4495278	4495483
158	l(3)10615	em_common_peak-3644	chr3R	5243520	5243916
159	ps	em_common_peak-3647	chr3R	5262301	5262926
160	CG31352	em_common_peak-3667	chr3R	5530902	5531288
161	CG11870	em_common_peak-3691	chr3R	6089874	6090275
162	pros	em_common_peak-3759	chr3R	7198347	7198779
163	CG14709	em_common_peak-3766	chr3R	7394869	7395200
164	Lk6	em_common_peak-3783	chr3R	7589428	7590085
165	Hsp70Aa	em_common_peak-3793	chr3R	7779883	7782306
166	Hsp70Ab	em_common_peak-3794	chr3R	7784220	7786637
167	Hsp70Ba	em_common_peak-3814	chr3R	8291280	8293650
168	Hsp70Bbb	em_common_peak-3815	chr3R	8328237	8330472
169	Hsp70Bb	em_common_peak-3816	chr3R	8331501	8333761
170	Hsp70Bc	em_common_peak-3817	chr3R	8334701	8337079
171	CG8790	em_common_peak-3849	chr3R	9190527	9190994
172	CG7886	em_common_peak-3945	chr3R	10468317	10468714
173	Tm1	em_common_peak-3991	chr3R	11116727	11117658
174	CG6006	em_common_peak-4032	chr3R	11983732	11984140
175	Abd-B	em_common_peak-4063	chr3R	12759784	12760282

176	Dad	em_common_peak-4067	chr3R	12879526	12879970
177	gukh	em_common_peak-4181	chr3R	14810014	14810757
178	CG6231	em_common_peak-4219	chr3R	15446569	15446976
179	bnl	em_common_peak-4232	chr3R	15662337	15662744
180	CG17273	em_common_peak-4260	chr3R	16655712	16656469
181	Atpalpha	em_common_peak-4267	chr3R	16783300	16783787
182	slou	em_common_peak-4287	chr3R	17382947	17383494
183	glec	em_common_peak-4309	chr3R	17681754	17682148
184	CG5346	em_common_peak-4356	chr3R	18354087	18354444
185	pnt	em_common_peak-4407	chr3R	19120859	19121294
186	CG31145	em_common_peak-4436	chr3R	19495135	19495557
187	Pli	em_common_peak-4447	chr3R	19715504	19715825
188	Gdh	em_common_peak-4452	chr3R	19768321	19768751
189	Hsp68	em_common_peak-4459	chr3R	19880371	19883020
190	CG17786	em_common_peak-4472	chr3R	20044226	20044718
191	jar	em_common_peak-4476	chr3R	20096675	20096993
192	CG6356	em_common_peak-4477	chr3R	20109464	20109916
193	CG5789	em_common_peak-4492	chr3R	20369431	20369686
194	CG5807	em_common_peak-4508	chr3R	20639605	20639938
195	CG31121	em_common_peak-4511	chr3R	20719833	20720231
196	CG11069	em_common_peak-4511	chr3R	20719833	20720231
197	fd96Cb	em_common_peak-4525	chr3R	20920602	20920878
198	Fur1	em_common_peak-4540	chr3R	21298531	21298859
199	LpR2	em_common_peak-4549	chr3R	21565894	21566251
200	Tsp96F	em_common_peak-4556	chr3R	21706863	21707151
201	HLHmbeta	em_common_peak-4559	chr3R	21830840	21831909
202	dei	em_common_peak-4579	chr3R	22266205	22266872
203	CG5455	em_common_peak-4582	chr3R	22287106	22287437
204	Tl	em_common_peak-4607	chr3R	22624665	22625065
205	T48	em_common_peak-4615	chr3R	22707402	22707709
206	Apc	em_common_peak-4721	chr3R	24670605	24670819
207	CG14509	em_common_peak-4738	chr3R	25020161	25020604
208	SP1029	em_common_peak-4741	chr3R	25075851	25076184
209	CG9743	em_common_peak-4792	chr3R	26028448	26028843
210	CG31012	em_common_peak-4825	chr3R	26634209	26635003
211	fz3	em_common_peak-4925	chrX	663990	664303
212	dnc	em_common_peak-5160	chrX	3150038	3150880
213	AlstR	em_common_peak-5246	chrX	3468491	3468786
214	CG32781	em_common_peak-5280	chrX	3642827	3643174
215	peb	em_common_peak-5402	chrX	4511357	4511706
216	ctp	em_common_peak-5409	chrX	4582178	4582493
217	CG6986	em_common_peak-5413	chrX	4601868	4602160
218	ovo	em_common_peak-5480	chrX	4942345	4942895
219	CG15465	em_common_peak-5511	chrX	5068787	5069268
220	CG3097	em_common_peak-5599	chrX	5664466	5664941

221	CG15765	em_common_peak-5602	chrX	5712472	5712922
222	Grip	em_common_peak-5621	chrX	5860278	5860676
223	l(1)G0030	em_common_peak-5651	chrX	6246258	6246613
224	CG3960	em_common_peak-5684	chrX	6465794	6466153
225	CG4615	em_common_peak-5740	chrX	6995936	6996330
226	Tbh	em_common_peak-5848	chrX	7889634	7890079
227	CG32711	em_common_peak-5908	chrX	8303720	8304078
228	Nrg	em_common_peak-5924	chrX	8411322	8411967
229	CG12075	em_common_peak-5984	chrX	8757867	8758360
230	CG15316	em_common_peak-6087	chrX	9393607	9394093
231	CG32700	em_common_peak-6095	chrX	9445014	9445370
232	l(1)G0320	em_common_peak-6096	chrX	9447436	9447833
233	Hex-A	em_common_peak-6097	chrX	9479973	9480393
234	spri	em_common_peak-6228	chrX	10478997	10479638
235	CG32676	em_common_peak-6243	chrX	10638664	10639047
236	ras	em_common_peak-6243	chrX	10638664	10639047
237	Ptp10D	em_common_peak-6330	chrX	11515887	11516280
238	fw	em_common_peak-6364	chrX	11897420	11898169
239	Tis11	em_common_peak-6451	chrX	12553395	12553726
240	CG15745	em_common_peak-6519	chrX	13199436	13199799
241	l(1)G0469	em_common_peak-6653	chrX	14105156	14105575
242	NetB	em_common_peak-6724	chrX	14643128	14643505
243	rut	em_common_peak-6735	chrX	14719496	14720158
244	Lsd-2	em_common_peak-6744	chrX	14969679	14970324
245	CG9095	em_common_peak-6755	chrX	15056479	15057308
246	l(1)G0168	em_common_peak-6785	chrX	15393195	15393663
247	sog	em_common_peak-6796	chrX	15520215	15520757
248	sd	em_common_peak-6809	chrX	15706331	15706888
249	Tob	em_common_peak-6827	chrX	15984782	15985185
250	CG9216	em_common_peak-6830	chrX	15998342	15998835
251	CG4829	em_common_peak-6906	chrX	16711023	16711728
252	wupA	em_common_peak-7021	chrX	18010579	18010902
253	l(1)G0003	em_common_peak-7071	chrX	18540975	18541393
254	CG32541	em_common_peak-7088	chrX	18824334	18824753
255	CG8062	em_common_peak-7136	chrX	19215856	19216292
256	kek5	em_common_peak-7140	chrX	19246777	19247456
257	CG14207	em_common_peak-7168	chrX	19499128	19499526

Supplementary Table 3. SSRs in MAR sequences

SSR	Genomic occurrances	Expected occurrance *	Actual occurrance	% as MAR	Fold Enrichment
**A**	**15617**	**407.6**	**4573**	**29.3**	**11.2**
**AC**	**9075**	**236.9**	**581**	**6.4**	**2.5**
**AGC**	**4804**	**125.4**	**364**	**7.6**	**2.9**
**AAC**	**2493**	**65.1**	**165**	**6.6**	**2.5**
**AG**	**2279**	**59.5**	**366**	**16.1**	**6.2**
**C**	**2025**	**52.9**	**558**	**27.6**	**10.6**
**AAAAAT**	**1380**	**36.0**	**124**	**9.0**	**3.4**
**AAAAAC**	**1147**	**29.9**	**126**	**11.0**	**4.2**
**AACAGC**	**1041**	**27.2**	**62**	**6.0**	**2.3**
**AAAC**	**1013**	**26.4**	**56**	**5.5**	**2.1**
ACC	868	22.7	63	7.3	2.8
AGG	805	21.0	53	6.6	2.5
AAG	619	16.2	34	5.5	2.1
AAAATG	617	16.1	41	6.6	2.5
AAAAAG	496	12.9	86	17.3	6.6
CCG	444	11.6	25	5.6	2.2
AAAAGC	425	11.1	27	6.4	2.4
ACG	402	10.5	27	6.7	2.6
AAAACC	367	9.6	24	6.5	2.5
AACC	355	9.3	19	5.4	2.1
AAACTG	345	9.0	18	5.2	2.0
AAAGCC	304	7.9	16	5.3	2.0
AAAG	295	7.7	27	9.2	3.5
AAAACG	248	6.5	18	7.3	2.8
AAACCG	232	6.1	13	5.6	2.1
AGGGGG	230	6.0	31	13.5	5.2
AAAAT	224	5.8	37	16.5	6.3
AAAAGG	216	5.6	33	15.3	5.9
ACACTC	215	5.6	14	6.5	2.5
AAGGAG	203	5.3	21	10.3	4.0
AGCGGC	201	5.2	11	5.5	2.1
AAAGAG	198	5.2	21	10.6	4.1
ACCC	190	5.0	11	5.8	2.2
AAGCAG	188	4.9	11	5.9	2.2
ACACCC	172	4.5	15	8.7	3.3
AAACCC	169	4.4	14	8.3	3.2

AACTCC	162	4.2	9	5.6	2.1
AAAGGC	160	4.2	10	6.3	2.4
AATCG	155	4.0	9	5.8	2.2
AAAAC	151	3.9	39	25.8	9.9
ACCAGC	151	3.9	8	5.3	2.0
AACTG	139	3.6	11	7.9	3.0
AAACG	136	3.5	14	10.3	3.9
AAACC	132	3.4	24	18.2	7.0
AGAGCG	131	3.4	12	9.2	3.5
AATAGC	130	3.4	9	6.9	2.7
ACGTCG	121	3.2	10	8.3	3.2
ACAGAG	119	3.1	9	7.6	2.9
ACCCCC	116	3.0	11	9.5	3.6
AACACC	114	3.0	8	7.0	2.7
ACCG	112	2.9	7	6.3	2.4
AAAGGG	111	2.9	14	12.6	4.8
AATCGC	109	2.8	6	5.5	2.1
AACG	107	2.8	6	5.6	2.1
AACCAC	106	2.8	18	17.0	6.5
AGCG	106	2.8	12	11.3	4.3
AACGGC	105	2.7	7	6.7	2.6

* Expected occurrance has been calculated based on the observation that 2.61% of total genomic DNA is MAR.

Supplementary Table 4. Primers used for amplification of MARs

Lane 1	CHR2L-F-1	GACAACAGCAGTCACGGTTC
	CHR2L-R-1	CGTCTGGCTAAGTGGTGTGA
Lane 2	CHR2L-F-2	ACACTGTCAGAATCCCGCTC
	CHR2L-R-2	AAAGCAAAGCTGCGAGTTTC
Lane 3	CHR2L-F-3	GCGTTTGAAATGGTTTCGTT
	CHR2L-R-3	TCTGTTCGGTTCTGTAGTGCAT
Lane 4	CHR2L-F-4	TTTGATTCCTTAGTAAGTTTTCAGAAT
	CHR2L-R-4	TTCAGCTTGATTTTGCTATTTTT
Lane 5	CHR2L-F-5	ATCTTCCTCGGGTCTTTGCT
	CHR2L-R-5	AAACGGTTATCGCTCGCTC
Lane 6	CHR2R-F-1	GGAGGCTTCTTGCGATTCTA
	CHR2R-R-1	GGCGACGACGTTAGTACCTC
Lane 7	CHR2R-F-2	TTTTTCCACGTCAAACCACA
	CHR2R-R-2	TCATTATGGCCCAAGGAAAG
Lane 8	CHR2R-F-3	GGAGCTGGGTTCTTCTTCCT
	CHR2R-R-3	CAAGGCAGTGATAGCGACAG
Lane 9	CHR2R-F-4	TTCAGCCATGTGCCTAAAAA
	CHR2R-R-4	GAGAGCAAAAGGTGACCAGC
Lane 10	CHR2R-F-5	GACGGACTGGTGGATGACTT
	CHR2R-R-5	CAAACATAACACGACCGACG
Lane 11	CHR2R-F-6	ACATTTTGAGCAGCTCCAGG
	CHR2R-R-6	ACGACGACGTTGTCCTAAGC
Lane 12	CHR3L-F-1	TATCCTTCAGCAGCGTCCTT
	CHR3L-R-1	ACATTCCTTTTTGGTGCAGG
Lane 13	CHR3L-F-2	GGCTGGATCACAACACACAC
	CHR3L-R-2	AAACGTTCAGTTCGCTTTGC
Lane 14	CHR3L-F-3	GGGCAATTGTTGATAATATCGTG
	CHR3L-R-3	AGGGTGTTTTCTTAATACCTTTAG
Lane 15	CHR3R-F-1	AATGCAGCAGGCAGTTTTCT
	CHR3R-R-1	TCTTTCAAAATCCGCAGGTC
Lane 16	CHR3R-F-2	TAGATCCGCCATCGATTCTT
	CHR3R-R-2	GGTAAAAGAGCATCTAGACTTCAACC
Lane 17	CHR4-F-1	TAGTGTGTGACCGTTTTGGC
	CHR4-R-1	CTAACACCCACAAACCACCC
Lane 18	CHR4-F-2	CCTTCATTATATAAGTTTTTCGGTCTG
	CHR4-R-2	TTGGGATGTTTTGCTGTTTTC
Lane 19	CHRX-F-1	AGCTCTGCTAGCACAACCACT

	CHRX-R-1	TCGACTGGCCTTTAGATGCT
Lane 20	CHRX-F-3	TTCCATTTGGAACCTGTCAAG
	CHRX-R-3	GCACTCTGGGTCACGGTATT
Lane 21	CHRX-F-4	CAGTCTGAGGCTGTTGATCG
	CHRX-R-4	AAAAATTGGAACGCACGTC
Lane C1	CONT-F-1	GCAGCGTACATCGACTTGAA
	CONT-R-1	GCAGAAGAGCAACCGAAGTT
Lane C2	CONT-F-2	CAATGTCTCTCGATCTGGCA
	CONT-R-2	ATTATGGCCACTTTTGGCTG
Lane C3	CONT-F-3	TTTAACATCACTTGCCGCAG
	CONT-R-3	CCAGAAATCCTAGTTCGCCA
Lane C4	CONT-F-4	TGTAAATTTTGTCCAAAGGGC
	CONT-R-4	ACATTGAATCGTTGGAAGGC
Lane C5	CONT-F-5	ACTCTGCACTTTGGCGTCTT
	CONT-R-5	CAGCACTTGTCCCAAGATGA
Lane C6	CONT-F-6	ACCACACGAGCCCAATTAAC
	CONT-R-6	TTCTGATTGCCAAGGCTTTT
Lane C7	CONT-F-7	AATTTTCCACTTTCTGCTGCTC
	CONT-R-7	TCAGCAAAGCTCCTTTTTCC
Lane C8	CONT-F-8	TTGTTTTCCTTTATTGCCCA
	CONT-R-8	GGAGTGGGTGATGAAAGGC
